# Mathematical Modeling of the Release of Active Ingredients from a Contraceptive Patch: Ortho Evra^®^ as a Case Study

**Published:** 2014

**Authors:** Grissel Trujillo-de Santiago, Carlos Patricio Sáenz-Collins, Lizette García-Arellano, Mario Moisés Álvarez

**Affiliations:** FEMSA-Biotechnology Center, Monterrey Tech (Centro de Biotecnología-FEMSA, Tecnológico de Monterrey). Ave. Eugenio Garza Sada 2501 sur. Col. Tecnológico, Monterrey, Nuevo León, México.C.P.64849.

**Keywords:** Contraceptive patch, Modeling, Norelgestromin, Ethinylestradiol, Pharmacodynamics

## Abstract

Contraceptive patches have become a frequently used contraceptive method. We present a mathematical model that describes the serum concentration profiles of Norelgestromin (NGMN) and Ethinylestradiol (EE) released from the contraceptive patch Ortho Evra^®^.

We propose a simple one-compartment model based on pharmacokinetics data reported in previous studies. The model assumes a time-dependent release rate and a first order elimination rate for each of the active ingredients contained in the patch. The model was applied to noncompliance scenarios, such as total and partial detachment of the patch or prolonged use without patch replacement. The proposed model adequately describes the clinically observed evolution of NGMN and EE in serum. Predictions from the model were successfully validated using reported experimental data of serum concentrations of NGMN and EE.

This simple model can be a valuable tool to predict pharmacokinetic profiles in diverse scenarios such us non-compliance situations. Alternatively, the model can be conveniently adapted to anticipate the effect of variations on patch characteristics such as differences in contact area, doses, materials, among others.

## Introduction

Transdermal delivery systems, as a route for administration of drugs, offer a number of advantages over the oral route (1,2). Namely, 1) they provide a constant and prolonged release of the drug, thereby maintaining a therapeutically effective concentration; 2) they avoid degradation of active substances and difficulties in absorption during gastrointestinal tract transit; 3) their application is easy and painless; and 4) patient compliance is significantly higher (1,2). 

The pharmaceutical market now offers a wide variety of drugs formulated for administration as a patch, including several related to hormone delivery (3-6,8,9). In particular, the Ortho Evra® contraceptive patch has a significant presence in the market. More than 5 million women have used this birth control patch since it was commercially introduced in 2002. In 2004, sales reached 411 million dollars (7). 

The Ortho Evra® contraceptive patch is a transdermal delivery system with a contact surface area of 20 cm^2 ^(8). The device contains 6 mg of Norelgestromin (NGMN) and 0.6 mg of Ethinylestradiol (EE) and releases 150 μg/d and 20 μg/d, respectively, into systemic circulation. The treatment consists of applying one patch per week over a three week (21 day) period, followed by a recess (8). After patch application, NGMN and EE appear in the plasma almost immediately, reaching a concentration plateau after 48 h, and then maintaining a steady state concentration (C^ss^) throughout the entire period of use. Once in the plasma, some NGMN liver metabolism occurs, resulting in the formation of compounds that include norgestrel and various hydroxylated and conjugated metabolites. The EE is also metabolized into various hydroxylated products and their glucuronide and sulfate conjugates. NGMN and EE metabolites are eliminated by renal and fecal routes (8).

In terms of its construction, the patch has three layers. The backing layer provides structural support and protects the middle layer from the external environment. The middle layer contains inactive compounds, including adhesive and the active ingredients, the hormones NGMN and EE. The third layer is a transparent film that protects the adhesive during storage; it is removed just before application (6). Previous studies have shown that, regardless of the application site (abdomen, buttocks, shoulder, or torso [excluding breasts]), and even under conditions of heat, humidity, exercise, and immersion in cold water, the patch results in effective concentrations of NGMN and EE in the serum (6,8-10).

This transdermal delivery system offers many advantages over oral administration (11). The continuous delivery of the drug through the skin allows stable plasma levels and avoids the fluctuating concentrations that occur with oral administration. In addition, the use of the patch avoids the gastrointestinal tract (and therefore enzymatic degradation and absorption difficulties within), which which allows reduced doses. The controlled release of active ingredients can also minimize systemic side effects, particularly those associated with high levels of drugs in the plasma. In addition, the “once-a-week” dose regimen significantly facilitates treatment compliance (11), as widely documented in recent reports (12-14). In general users express a higher level of satisfaction with the contraceptive patch than with oral contraceptives (14). Despite this proven superiority of compliance versus oral contraceptive options (90% versus 80%), contraceptive patches users still fail to follow perfect cycle recommendations in 10% of the cases. This behavior is consistent among different age groups (12,13). Although patch adhesive properties have proven to be very good among different climate conditions, still in approximately 5% of applications, patches are susceptible to detach at least partially (10). The consequences of different degrees of compliance deviations on contraceptive efficacy are difficult to estimate experimentally and only retrospective data is available (10-14). In addition, some controversy has recently arise on the increased risk of venous thromboembolism in contraceptive patches users (15-17). Mathematical modeling can be a useful tool to study the effects of different non-compliance scenarios. The use of simple but reliable mathematical models might also provide an additional tool to rationally improve contraceptive patch design (in terms of doses or materials) to fine-tune the window of effective but safe concentrations of the active ingredients in serum. Unfortunately, there are only a very limited number of contributions in the area of mathematical modeling of transdermal delivery systems (18-21). Most of them focus on presenting the conceptual and mathematical framework (18) to understand and construct mathematical models for the description and design of traditional (18-20) and novel transdermal systems (21). To our knowledge, there are no reports available on the use of modeling for actual clinical forecast of commercial contraceptive patches. In this contribution, we present an extremely simple mathematical model capable of accurately describing existing pharmacokinetic data related to serum concentration curves of NGMN and EE in the serum of volunteers administered with Ortho Evra® (8,6). The proposed model is then used to estimate the effect of several possible non-compliance scenarios including those related to partial or full patch detachment.

## Experimental

**Figure 1:**
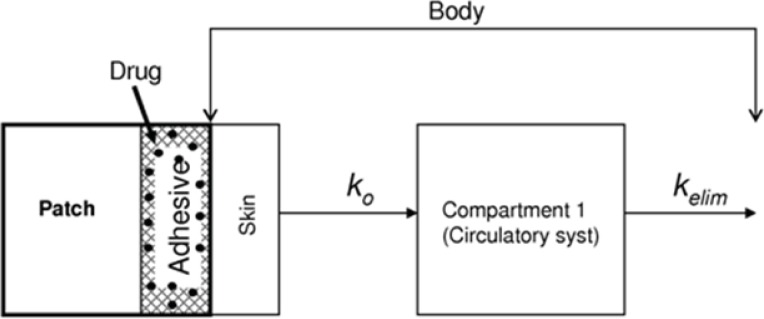
Schematic representation of a one-compartment mathematical model to simulate the phamacodynamics of Norelgestromin (NGMN) and Ethinylestradiol (EE) released from a contraceptive patch. Entry arrows represent the release of the active ingredients from the patch; exit arrows represent elimination from the blood stream

In this contribution, we present a mathematical model for simulation of the kinetics of NGMN and EE release into the serum from contraceptive patches. The model considers only one compartment, the blood plasma. Figure 1 presents a descriptive diagram of the pharmacokinetics of the active compounds of the contraceptive patch, where k_o_ is a proportionality constant reflecting the amount of drug that enters the blood stream and K_elim_ is the corresponding elimination constant. 

Therefore, it is considered that two competing processes contribute to the accumulation of each component in blood plasma: release from the contraceptive patch into the blood stream (absorption) and metabolic elimination from the blood stream (elimination). Absorption is considered the only stream contributing positively to the accumulation of each active ingredient in the main compartment. Consequently, absorption rates are defined for each active ingredient (R_abs NGMN_ and R_abs EE_). Elimination is conceptually an outlet contributing negatively to the overall accumulation rate of NGMN and EE in the main compartment. Elimination rates are defined for each active ingredient (R_elim NGMN_ and R_elim EE_). 

d[NGMN]/dt= R_abs NGMN_ – R_elim NGMN_                    Equation (1a)

d[EE]/dt= R_abs EE_ – R_elim EE_                     Equation (1b)

Here, [NGMN] and [EE] are the concentrations of NGMN and EE respectively, and t is time. 

The elimination rate of each active ingredient from the bloodstream (R_elim NGMN_ and R_elim EE_ ) was considered to follow a first order decay kinetics:

 R_elim NGMN_ =[NGMN]_elim_/**Δ**t = - k_elim N_ [NGMN]                      Equation (2a)

R_elim EE_ =**Δ** [EE]_elim_/**Δ**t = - k_elim E_ [EE]                      Equation (2b)

Due to the discrete nature of the clinical data used, it is convenient to express these differential equations as equations of differences. Then, 

**Δ**[NGMN]_elim_= -k_elim N _ [NGMN] **Δ**t                     Equation (3a)

**Δ**[EE]_elim_= -k_elim E _ [EE] **Δ**t                     Equation (3b)

where k_elim_ is the first-order elimination constant, **Δ**[NGMN] y **Δ**[EE] are the changes in plasma concentrations of NGMN and EE, respectively, and **Δ**t is the change in time.

The entry of the drug into systemic circulation was defined as a time-dependent function. In agreement with experimental data reported by Abrams *et al.* (8,6), we assumed that the variation in the entry rate of each drug follows an exponential function in time. Equations 4a and 4b describe the process of absorption of each drug in the bloodstream:

R_abs NGMN_ = **Δ**[NGMN]_abs_/**Δ**t = (k_o N_/V_d N_) exp{-αt}                     Equation (4a)

R_abs EE_ = **Δ**[EE]_abs_/**Δ**t = (k_o E_/V_d E_) exp{-βt}                     Equation (4b)

Therefore, expressed as equations of difference:

**Δ**[NGMN]_abs_= (k_o N_ exp{-αt}/V_d N_) **Δ**t                     Equation (5a)

**Δ**[EE]_abs_= (k_o E_ exp{-βt}/V_d E_) **Δ**t                     Equation (5b)

Where k_o_ represents the rate of release of each drug at time zero, α and β are constants that determine the rate at which the active compounds are absorbed into the blood, and V_d_ is the volume of the distribution of the drugs, which can be calculated by dividing the clearance by the corresponding k_elim_ (22).

 Equations 6a and 6b model the kinetics of plasma concentration for NGMN and EE:

**Δ**[NGMN]= (k_o N_ exp{-αt}/V_d N _- k_elim N_ [NGMN]) **Δ**t                     Equation (6a)

**Δ**[EE]= (k_o E_ exp{-βt}/V_d N _- k_elim E_ [EE]) **Δ**t                     Equation (6b)

The k_o_ is considered to reset each time the patch is replaced.

*Model validation*

The model was validated by assessment of graphical adjustment between simulated and experimentally obtained curves of NGMN and EE serum concentration curves, and by comparison with pharmacokinetic parameter values reported in the literature (6, 8,23-25). Mainly, experimental curves of plasma concentration for NGMN and EE after once a week application of Ortho Evra® (according to the manufacturer recommendation) were used (6,8). Other relevant pharmacodynamic data, including the average steady state concentration (C^ss^) throughout the entire period of use, average doses absorbed into the circulation system, and average half-life of each active ingredient were taken from pharmaceutical Handbooks (24,25).

## Results and Discussion

*Model results versus reported pharmacokinetic data*

**Figure 2:**
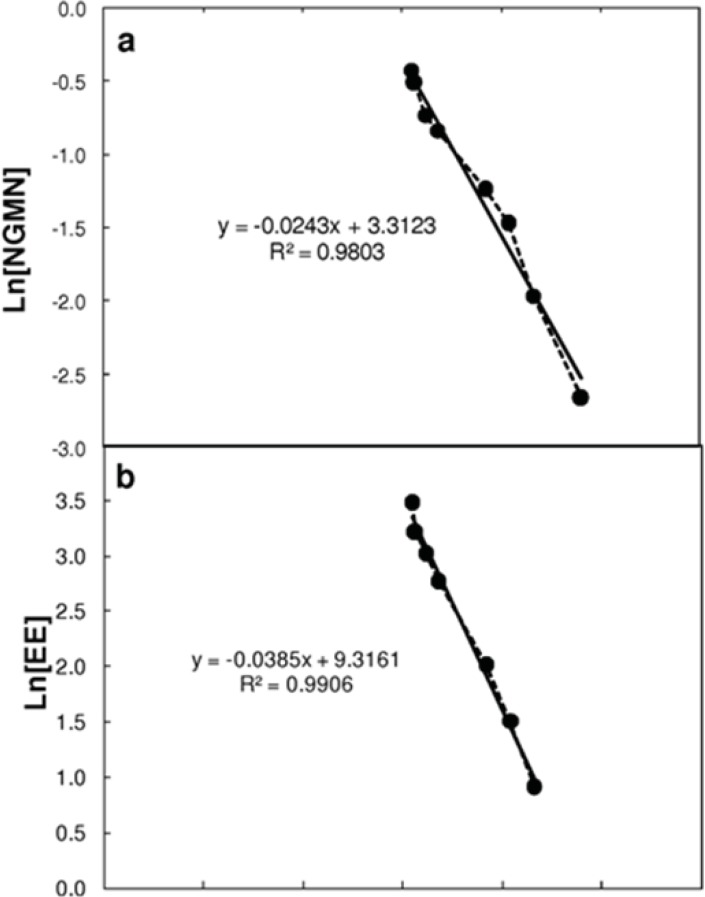
Estimation of the elimination constant (K_elim_) by linearization of the concentration profiles of (a) Norelgestromin (NGMN) and (b) Ethinylestradiol (EE) in plasma after patch removal. Linearization was performed by assuming a first order decay, and plotting the natural logarithm of the concentration of each active ingredient versus time. The slope of each curve corresponds to the elimination rate constant

Table 1 shows the pharmacokinetic parameter values considered for solution of the model. Most parameters were taken or calculated from data reported by Abrams *et al.* (6,8). The elimination constant (K_elim_) was obtained by plotting the natural logarithm of the plasma concentration values for NGMN and EE after patch removal at the end of the cycle. The elimination process followed an exponential decay behavior that was a first order process (dependent on the drug concentration in the serum) characterized by a single constant (see Figure 2).

**Table 1:** Pharmacokinetic values of Norelgestromin (NGMN) and Ethinylestradiol (EE). Obtained or calculated from Abrams *et al*. (8).

**Kinetic constants **	**NGMN**	**EE**
***k***_o_	150 μg day^-1^	20.5 μg day^-1^
***Cl *** ^a^	7.89 L h^-1^	18.3 L h^-1^
***t***_1/2 _^b^	28.4 h	15.2 h
***k***_elim _^c^	0.0244 h^-1^	0.0385 h^-1^
***V***_d _^d^	323,300 mL	401,385 mL
***α***	0.0028	
***β***		0.0034

^a^ *Cl Drug clearance*

^b^ *t*_1/2_ *: half-life*

^c ^k_elim_= Ln2/t_1/2_.

^d ^V_d_ = Cl / k_elim_

Probably the most adventurous assumption made in our model refers to the time dependence of the absorption rates of each active ingredient. We proposed that the variation in absorption rate with time corresponded to an exponential function (see Equations 5a and 5b). Ideally, a descriptive model of release/absorption should consider (a) diffusion of the active compound from the bulk of the patch to its surface, (b) diffusion of the active compound from the membrane of the patch to the *stratum corneum* of the skin, (c) sorption and penetration from the *stratum corneum* to inner layers of the tissue, and (d) uptake from the capillary network (19,20). It has been suggested that either the *stratum corneum* (19) or the delivery device can control the rate of drug delivery to plasma (20). Moreover, the observed flux can be the result of the combined effects of the skin and the device (20). In the model presented here, we follow the later approach and consider that the combined effects of the transport through the patch and the skin can be modeled by the actual delivery rate (k_o_) and an exponential decay component of the type [exp (-α*t)]. This exponential decay term conceptually lumps the effect of diverse mechanisms responsible of progressively attenuating the release of the active compound in time (depletion of the active compound from the bulk of the patch, saturation of the *stratum corneum*, changes in the permeability of the patch due to use, among others).

**Figure 3:**
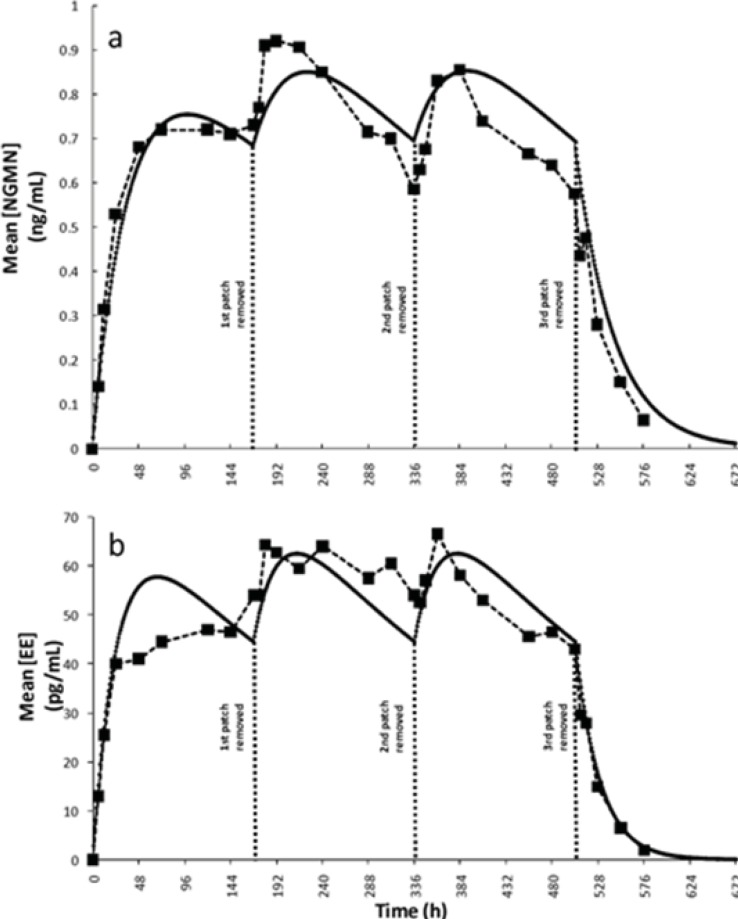
Simulation results and validation of the model. Plasma concentration profiles for a typical three weeks administration treatment: (a) Norelgestrmin (NGMN) obtained by Abrams *et al*. (6) (--■--), as predicted by simulations (—). (b) Ethinylestradiol (EE) experimentally obtained by Abrams *et al*. (6) (--■--), as predicted by simulations (—).

In literature, k_o_ values of 150 ± 38.0 and 20.5 ± 6.6 were originally reported (6) for NGMN and EE, respectively. Recently, Health Canada endorsed a bulletin by Janssen-Ortho (27), manufacturer of Ortho-Evra, providing updated information on the average daily release rates of NGMN (200 micrograms [μg] every 24 h) and EE (35 μg every 24 h). These updated values were used in the model as initial absorption rates. The parameters α and β, modulating the time dependency of the overall absorption rate for each active ingredient, were adjusted using the experimental data presented by Abrams *et al.*(8). Indeed α and β were the only two adjustable parameters used in the model. We observed a significant improvement in the fitting to experimental data (measured as mean squared error) when this assumption of time dependant absorption rate was implemented. Figures 3a and 3b show comparisons of the concentration profiles obtained from simulations using the proposed model with the experimental data reported previously by Abrams *et al.*(8). This simulation corresponded to a typical treatment. The periodic behavior displayed was due to the restoration of the k_o_ when the patch was replaced at the start of weeks 2 and 3. The literature data also indicated that plateau serum concentrations for both active principles are typically reached approximately 48 h after the patch has been applied (24, 25). A consistent behavior was observed in our simulation results (see Figure 3).

We also compared several relevant pharmacokinetic parameters, including the steady state concentration of each active ingredient (C^ss^), the half-life time (t_½_), and the area under the curve of plasma concentration versus time per week (AUC_0-168h_). As shown in Table 2, values derived from modeling showed a satisfactory agreement with experimentally calculated values.

**Table 2:** Comparison of reference pharmacokinetic values (8, 9) and those obtained through the model

	**Reference values (8, 9)**	**Predicted values from model fit**
**NGMN**		
C^ss^(ng ml^-1^)^a^	0.83 ± 0.21	0.773
AUC_0__-168 _(ng h ml^-1^)^b^	123 ± 32.3	123.40
t_1/2 _(h)^c^	28.4 ± 12.8	27.96
k_0_ (μg day^-1^)	150 ± 38.0	150 ± 20.5
**EE**		
C^ss^ (pg ml^-1^)^a^	56.7± 22.6	52.63
AUC_0-168 _(pg h ml^-1^)^ b^	8543 ± 3488	8463.75
t_1/2 _(h)^ c^	15.2 ± 3.3	15.57
k_0_ (μg day^-1^)	20.5 ± 6.6	20,67 ± 3.41

C^ss^= Plasma concentration on the steady state; AUC_0-168_:=Area Under a Curve of plasma concentrations versus week time; t_1/2 _= Elimination half time

^a^ Concentrations average since the 48h; at this time the C_ss _has been reached.

^b ^AUC_0-168_ average in a tree weeks cycle

^c^ Time taken for the plasma concentration to fall to half its initial value at the moment to remove the patch.

In summary, the proposed mathematical model reproduces the experimentally observed pharmacokinetic behavior reported previously. To do so, the model assumes a restoration of k_o_ at each patch replacement, and a time-dependent rate of drug release into the plasma. The variation in the release rate is attributed to two factors: a gradual decrease in the hormone concentration in the patch, and a gradual saturation of the skin with the active ingredients. Both of these effects would decrease the rate of drug absorption. As the model shows, the efficacy of the contraceptive treatment is not severely compromised by these factors under a typical administration scheme.

Beyond describing the behavior of NGMN and EE in serum, the proposed mathematical model can be used to predict pharmacokinetic behaviors under different administration scenarios.

*Simulation of non-compliant scenarios*

Under the recommended administration protocol, the patch does not cause any overdose because the amounts of hormones released are adequately balanced by drug elimination. However, non-compliance is a common problem compromising treatment effectiveness. Particularly, in the case of contraceptive patches, the occurrence of non-compliance behavior has been relatively well characterized (12-14). For example, in a sample of 812 north american women using contraceptive patches, the percentage of perfect compliance range between 88.1% and 91% among different age groups (12). In another study, a population of 812 users of contraceptive patches exhibited perfect compliances in the range of 89.6 to 91.8%. In an parallel group of 605 users of oral contraceptives, perfect compliance did vary among different age brackets, being as low as 67.7% in users aged 18-20 years and as high as 80% in those aged 30 years and older (13). Although perfect compliance among patch users is comparatively high, the consequences of non-compliance events are highly significant. In that very same study, the risk of pregnancy increased 5 to 10 fold among the non-perfectly compliance subgroup (13).

Our model was also used to explore performance under common non-compliance scenarios, with the aim of estimating potential pregnancy risk or even suggesting adequate corrective actions.

**Figure 4:**
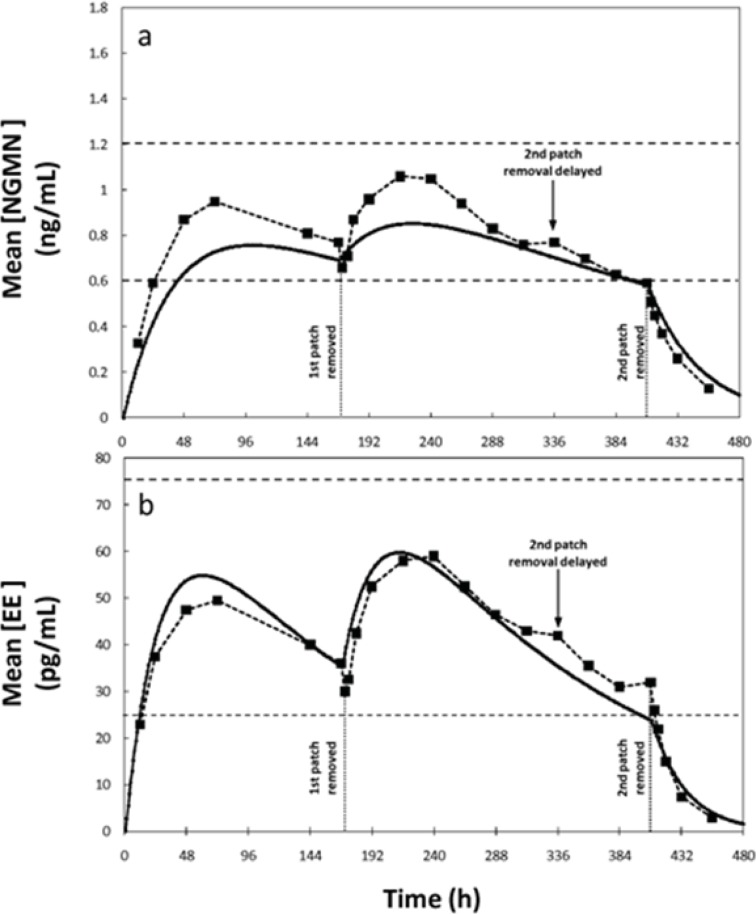
Concentration profiles of Norelgestromin (NGMN) and Ethinylestradiol (EE) for a non-compliance scenario where patch removal is delayed during the second week of application. Comparison 1of experimental data reported by Abrams *et al*. (6) (--■--), and (b) predictions obtained by simulations using the proposed model (—).

For example, for a patch administration regimen consisting of a 504 h cycle (3 weeks), with patch replacement every 168 h (7 days), effective concentration values of 0.6–1.2 ng/mL for NGMN and 25–75 pg/mL for EE have been established (6,8). For comparison, we considered an alternative scenario where the user forgets to replace the patch at the prescribed time (14^th^ day or 336 h), and retains it until the 17^th^ day. The experimentally determined concentration profiles for this scenario were reported elsewhere (8). Through blood sampling, the authors found that serum concentrations of NGMN and EE remain in the reference therapeutic range during these 17 days, suggesting that the treatment is still effective between day 14 and 17. Similarly, our simulation results indicated that concentration of EE remains within the therapeutic range despite the delayed patch replacement but the concentration of NGMN falls below the effective range at day 16 for approximately 26 hours (Figure 4). Note that, at hour 408, the serum concentrations of both hormones are at the lowest values of the reference range. Further delay in patch replacement would be detrimental to treatment effectiveness.

**Figure 5:**
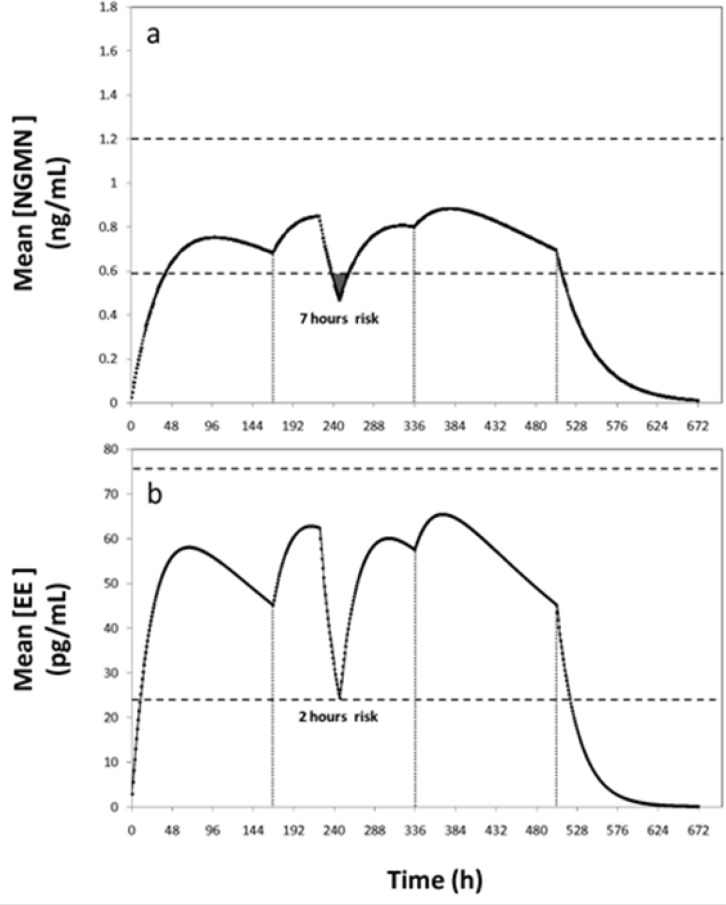
Concentration profiles of (a) Ethinylestradiol (EE) and (b) Norelgestromin (NGMN) for a non-compliance scenario where the patch is completely detached for 24 h during the second week of contraceptive treatment. Patch detachment and patch reapplication events are indicated with arrows. Therapeutic range is indicated between dashed lines

A second non-compliance case would be a patient who forgets for 1 or 2 days to replace the patch in the second or third week of the cycle. In these circumstances, the manufacturer recommends applying a new patch and continuing the cycle to maintain an acceptable low risk of pregnancy. If the patient forgets to change the patch for more than 2 days, the risk of conception rises and a new cycle should be started. Use of additional contraceptive methods is also recommended to reinforce protection during the first week of the new cycle. These indications are consistent with our simulation results. The efficacy of the contraceptive treatment becomes seriously compromised if the patch is not replaced after the second day of the second or third week of application (Figure 5).

**Figure 6:**
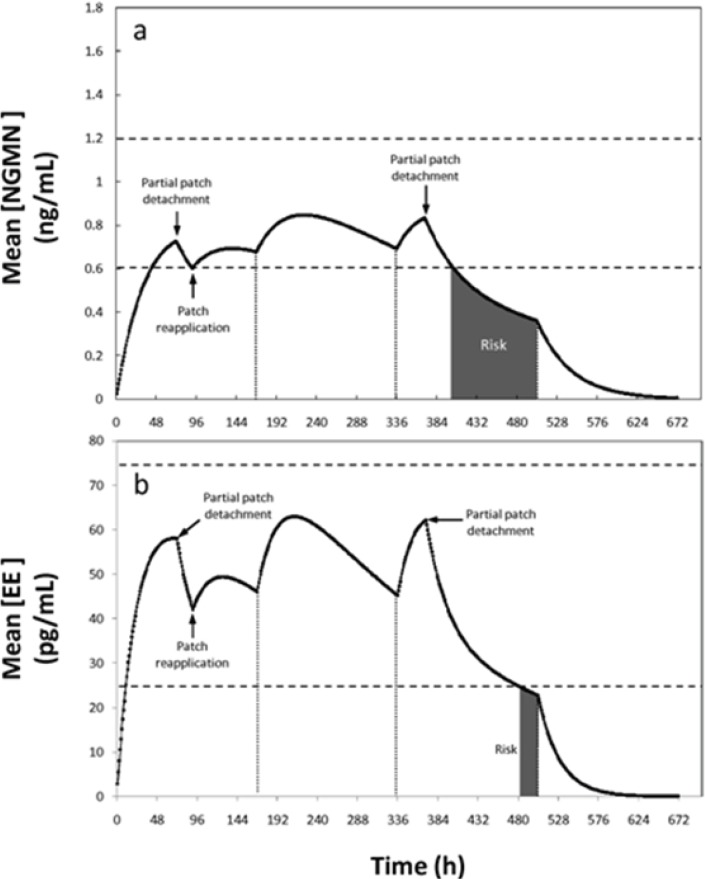
Concentration profiles of (a) Ethinylestradiol (EE) and (b) Norelgestromin (NGMN) for a non-compliance scenario where a patch is partially detached (50% of its contact area is detached for 24 hours) during the first and third week of contraceptive treatment. Patch detachment and patch reapplication events are indicated with arrows. Therapeutic range is indicated between dashed lines. Periods with pregnancy risk are indicated as shaded areas

In a third non-compliance situation, partial or complete patch detachment was simulated. In practice this is a relevant scenario to simulate. In a recent study with more than 3319 woman, the adhesion of Ortho Evra® under different climate and physical activity conditions (including exercising) was determined (10). Results indicated that patch adhesion is adequate and not statistically compromised by weather or activity conditions. Only nearly 5% of the Ortho Evra patches applied in the referred study were detached partially (2.9) or totally (1.8%) (10). Therefore, although not highly frequent, the event of patch detachment and its potential consequences in patch efficacy deserves some attention. In the event of total patch detachment, if the patch was absent for less than 24 h, the manufacturer recommends re-application in the same place or immediate application of a new patch, without any adjustment of the regimen. In particular, detachment of the patch occurring during the first week becomes a critical situation, considering that the serum concentrations of the patch active ingredients are at lower levels than during the following weeks. The proposed model can be used to study partial detachment situations in detail. For example, if only 50% of the patch area detaches at the end of day three of the first cycle, situation depicted in Figure 6, the concentration of NGMN becomes the limiting factor. Our simulations suggest that, following detachment, the concentrations remain within the therapeutic reference range for 22 h for NGMN (see Figure 6).

Therefore, based on the model output, a window of pregnancy risk begins 22 h after patch detachment. Replacement or re-application of the patch is therefore advisable within this timeframe. If the time limit of 22 h is exceeded, a new patch should be applied and a new cycle should be started. In addition, a backup contraception method should also be used for at least one week after detachment. This is, however, not the most pessimistic scenario, since at the end of the third day the concentration of NGMN has reached its plateau its first week plateau. Based on the model output, if a detachment of 50% of the total area of the patch occurs earlier during the first week (for example after 48 h), the device should be re-adhered within a period of no longer than 14 h to maintain efficacy (scenario not shown). Consistent with the current clinical recommendation, the regimen should not be changed if replacement is done during this time period. 

During the second and third weeks, when higher drug concentrations are achieved in the serum, this 22 h period could be extended to approximately 34 h without increasing the pregnancy risk (see Figure 6). Consequently, the model partially supports the manufacturer’s indications related to patch detachment scenarios. Our results suggest the establishment of specific indications for critical situations. Full patch detachment and the occurrence of incidents during the first week of application should be considered as particularly critical conditions.

It is important to note that the proposed model is not able to address all physiological events that occur due to treatment non-compliance. For example, after an intentional dosing error, significantly lower follicular size and incidence of ovulation have been reported in women using the Ortho EVRA® patch than in those who used oral contraceptives (26). This data suggests that, for some non-compliance scenarios, the potential pregnancy risk caused by treatment faults could be higher than our model indicates.

Alternatively, the model can be conveniently adapted to anticipate the effect of variations on patch characteristics such as differences in contact area (9), doses and materials with different properties. Recently, there has been controversy on the possible increased risk of venous thromboembolism (VTE) in contraceptive patches containing EE (15-17). Although some reports indicate that no-increased risk of VTE was observed in women using contraceptive patches versus oral formulations containing EE (15,16), other reports suggest an increased risk of 5 fold in subjects using Ortho Evra® (17) Mathematical modeling can be a valuable tool to determine optimal concentrations of active ingredients, or alternatively precise surface areas, that guarantee lower side effects without compromising efficacy. In our simple model, a sensibility study on the effect of changing the surface area of the patch or the concentration of each active ingredient on their serum concentration profiles can be easily done by only adjusting initial condition values. With relatively minor modifications and additional pharmacokinetic data, the model could be even adapted to describe similar transdermal drug delivery devices.

## Conclusions

In summary, our mathematical model describes the serum concentration profiles of the active ingredients present on the Ortho EVRA® contraceptive patch (norelgestromin and ethinylestradiol). This simple model considers only one compartment, a time-dependent absorption rate, and a first order elimination (dependent on the serum concentration of each active ingredient). The model was validated using experimentally obtained pharmacokinetics values reported in the literature. The model could be used to describe diverse non-compliance scenarios and to suggest corrective recommendations that would properly address them.

*Declaration of Interests*

We thankfully acknowledge the financial support of Tecnológico de Monterrey (seed fund CAT-122 and The Excellence Scholarship Program). We thank to CONACyT (Consejo Nacional de Ciencia y Tecnología) for the graduate student stipend scholarships provided to Grissel Trujillo-de Santiago, Carlos Patricio Sáenz-Collins and Lizette García-Arellano.
